# Announcing the 2017 *Pharmaceuticals* Travel Award for Young Post-Doctoral Researchers

**DOI:** 10.3390/ph10020048

**Published:** 2017-05-22

**Authors:** Jean Jacques Vanden Eynde

**Affiliations:** Editor-in-chief, MDPI AG, St. Alban-Anlage 66, 4052 Basel, Switzerland; jean-jacques.vandeneynde@ex.umons.ac.be; Tel.: +32-2-355-81-61

Last year, for the first time in its history, our Journal was able to offer a travel grant of 800 CHF to a young researcher in the field of medicinal chemistry. The grant was awarded to Dr. Zesergio Melo Jerez working in the Institute of Neurobiology at the Universidad Nacional Autónoma de México, Querétaro, México [1]. Further to the success of that initiative, the Editorial Office decided to renew the call in 2017 but limited candidates to post-doctoral fellows. Researchers from 14 countries (Austria, Belgium, Brazil, France, Germany, Italy, Poland, Portugal, Romania, Russia, Spain, Tunisia, United Kingdom, and United States of America) sent their applications. All files were of excellent quality and the work of the evaluation committee was, this year again, a difficult and uncomfortable task. Finally, the selected candidate was Dr. Diana Resende who is affiliated at the Faculty of Pharmacy of the University of Porto [2], Portugal. She will present an oral communication at the 20th European Symposium on Organic Chemistry (ESOC 2017) [3] that will be held in Cologne (Germany) during the month of July (2–6 July). Her talk will be entitled “Synthetic Strategies for the Preparation of Quinazoline Alkaloid Derivatives with Promising Biological Activities”. On behalf of the Evaluation Committee, the editorial staff, and all our readers, we sincerely congratulate the awardee.


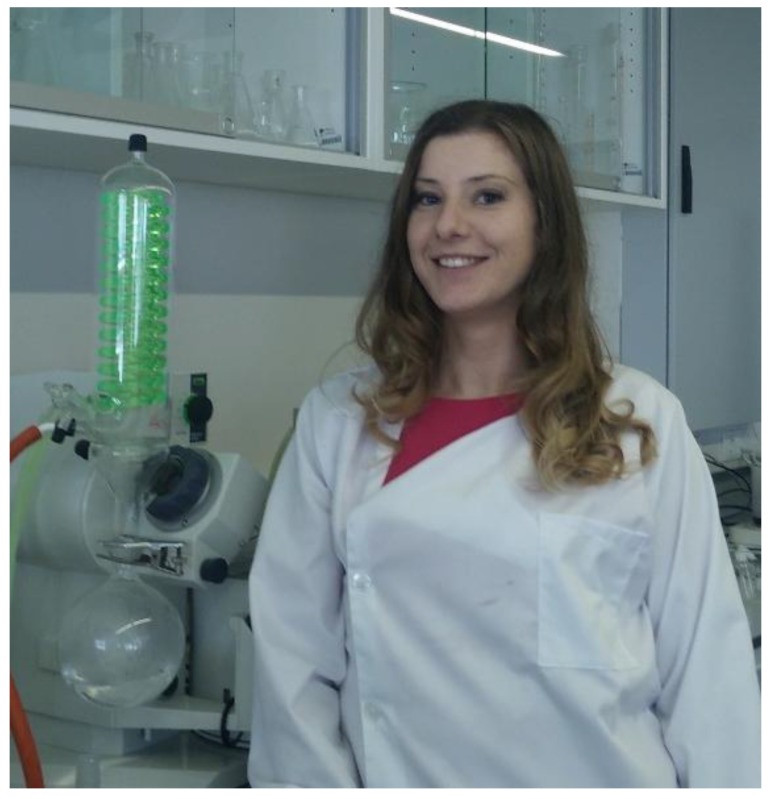


Diana Resende obtained her BSc in Chemistry from Aveiro University (Portugal) in 2007. She spent one year as Erasmus student at Prof. Antonio Mourino group at the University of Santiago de Compostela (Spain). During this period, she developed work in the design and synthesis of selective agonists for treatment of vitamin D-resistant rickets-associated vitamin D receptor mutations. After that, she joined the group of Professor Artur M. S. Silva as an MSc student. The work concerned organocatalytic asymmetric addition of nucleophiles to α,β,γ,δ-unsaturated compounds. She then proceeded her PhD studies working on synthesis of new heterocyclic compounds and cyclohexane derivatives through organocatalytic and metal-mediated transformations. In May 2016 she integrated CIIMAR (Interdisciplinary Centre of Marine and Environmental Research) as a postdoctoral fellow in the group of Prof. Madalena M. M. Pinto at the Laboratory of Organic and Pharmaceutical Chemistry of Faculty of Pharmacy, Porto (Portugal). Her current interests include synthesis of xanthone and fiscalin scaffolds derivatives based on marine natural models with promising biological activities.

We would like to thank all applicants for the high quality of their work. We wish them success in completing their research. Special acknowledgements are also dedicated to mentors of the applicants and to all of you who contributed to the success of the 2017 call of the *Pharmaceuticals* Travel Award for Young Researchers.
